# Calcium-Orchestrated Vascular Collapse in Cancer Therapy: Mechanisms, Nanotherapeutic Platforms, and Translational Perspectives

**DOI:** 10.3390/cancers18162592

**Published:** 2026-08-12

**Authors:** Fatima Zahra Kamal, Radu Lefter, Vasile Burlui, Alin Stelian Ciobîcă, Gabrielle Dăscălescu, Said Rammali, Andrei Luca, Ancuța Andreea Miler, Hina Alim, Otilia Novac, Bouchaib Bencharki, Bogdan Novac

**Affiliations:** 1 Care and Health Biology Team, 2S2D Laboratory, Higher Institute of Nursing Professions and Health Technical (ISPITS), Casablanca 20250, Morocco; fatimzahra.kamal@gmail.com; 2 Laboratory of Agro-Alimentary and Health, Faculty of Sciences and Techniques, Hassan First University of Settat, Settat 26000, Morocco; s.rammali@umi.ac.ma (S.R.); bencharki2003@yahoo.fr (B.B.); 3 Center of Biomedical Research, Romanian Academy, 700506 Iasi, Romania; radu_lefter@yahoo.com; 4 Department of Biology, Faculty of Biology, Alexandru Ioan Cuza University of Iasi, 700505 Iasi, Romania; secretariat@univapollonia.ro (V.B.); alin.ciobica@uaic.ro (A.S.C.); gabidascalescu2001@gmail.com (G.D.); 5 Ioan Hăulică Institute, Apollonia University of Iași, 700511 Iasi, Romania; 6 Olga Necrasov Center, Biomedical Research Group, Romanian Academy, 700481 Iasi, Romania; 7 CENEMED Platform for Interdisciplinary Research, Grigore T. Popa University of Medicine and Pharmacy, 700115 Iasi, Romania; 8 Human Nutrition, Bioacives and Oncogenetics Team, Faculty of Sciences, Moulay Ismail University, Meknes 11201, Morocco; 9 Faculty of Medicine, Carol Davila University of Medicine and Pharmacy, 020022 Bucharest, Romania; 10 Faculty of Medicine, Grigore T. Popa University of Medicine and Pharmacy, 700115 Iasi, Romania; mileranca@yahoo.com (A.A.M.); bogdannvc@gmail.com (B.N.); 11 Department of Life Sciences, University of Mumbai, Mumbai 400098, India; hina.alim@lifesciences.mu.ac.in

**Keywords:** calcium signaling, tumor vasculature, endothelial dysfunction, tumor microenvironment, nanomedicine, targeted drug delivery

## Abstract

Cancer therapy is increasingly focused on manipulating the tumor microenvironment rather than directly eradicating malignant cells. Vascular-targeting strategies are emerging, and calcium-mediated vascular disruption is an exciting approach through which rapid and irreversible blood flow shutdown can be achieved. Here, we overview the molecular and physiological basis of calcium signaling in vascular homeostasis and outline how unregulated calcium dysfunctions in endothelial cells compromise their functionality and represent therapeutic opportunities. Elevation of intracellular calcium concentrations in endothelial cells promotes their dysfunction, coagulation, mitochondrial collapse, oxidative stress, and ultimately apoptosis, resulting in catastrophic vascular depletion and secondary necrosis that follows such collapse. A promising area of calcium-mediated attack is the emergence of exciting nanotechnologies that result in the development of calcium phosphate, calcium carbonate, and calcium peroxide nanoparticles, exploiting the enhanced permeability and retention effect of nanoparticle therapeutics to achieve selective tumor accumulation and controlled calcium release. Indeed, hybrid therapeutic platforms that couple calcium dysregulation with chemotherapy, photodynamic therapy, sonodynamic therapy, immunotherapy, or thermal ablation can exhibit pronounced antitumor effects through synergistic means. There is good preclinical evidence for the feasibility of vascular collapse mediated via calcium dysregulation. The transition of calcium to the clinic faces hurdles in relation to biosafety, how to achieve precise delivery, pharmacokinetics, and regulatory harmonization. Compared to traditional vascular disrupting agents and anti-angiogenic therapies, calcium modalities can provide rapid occlusion of vessels, are less prone to resistance development, and potentially have less systemic toxicity. Overall, calcium-mediated vascular collapse is thus an exciting next-generation technology for the vascular-targeted treatment of cancer, and likely to play an important role in precision oncology therapeutics.

## Simple Summary

This review is focused on a new strategy for the treatment of cancer by calcium and calcium-based nanomaterials. Conventional cancer therapies face challenges such as drug resistance, limited targeting and harmful side effects. Recent advances in understanding the mechanism of tumor destruction by increased calcium in tumor blood vessels that rapidly disrupts tumor blood flow are reviewed. We also describe the synergistic effects of this approach when combined with other therapies. Early laboratory studies are promising, but the review also highlights the need for more testing to ensure it is safe and effective in people. This review synthesizes the current evidence and provides a comprehensive overview of the therapeutic promise and future clinical development of calcium-based therapies.

## 1. Introduction

Cancer therapy is increasingly shifting from tumor-centric approaches toward strategies that exploit vulnerabilities within the tumor microenvironment (TME) [1,2]. Among these, the abnormal tumor vasculature represents a key therapeutic target due to its structural and functional defects [3,4,5]. Vascular-targeting strategies aim to induce rapid shutdown of tumor blood flow, leading to nutrient deprivation and cell death [3]. Abnormal vasculature also contributes to treatment resistance by inducing hypoxia and elevated interstitial pressure [6]. Rather than relying solely on conventional vascular-targeting strategies, emerging approaches aim to exploit intrinsic vulnerabilities within tumor vasculature [1]. In this context, calcium dysregulation represents a critical and underutilized mechanism capable of inducing both cellular and vascular instability. By perturbing calcium homeostasis, it becomes possible to simultaneously disrupt endothelial integrity, impair tumor perfusion, and amplify downstream cytotoxic effects.

A particularly promising strategy is the exploitation of calcium-orchestrated vascular collapse, which hijacks the physiological signaling role of calcium to induce catastrophic failure of tumor blood vessels. Calcium signaling is a significant factor in tumor angiogenesis and vascular function; hence, it is an appealing route for cancer therapy [7,8]. Tumor endothelial cells exhibit altered calcium signaling and dysregulated expression of calcium channels than normal endothelial cells [7]. Targeting the tumor vasculature is intended to cause a rapid and catastrophic shutdown of blood flow, leading to tumor cells death due to the lack of oxygen and nutrients [3]. Calcium channels, including TRP and Orai families, regulate pro-angiogenic signaling in endothelial cells. Despite its central role in proliferation in cancer-associated pathways, including cell proliferation, and migration, as well as vascularization, few cancer drugs presently target calcium signaling machinery [8]. Targeting calcium signaling within tumor vasculature offers a selective and mechanistically distinct therapeutic strategy [8,9].

Here, we aim to review the current understanding of calcium signaling in tumor vascular biology and examine how therapeutic manipulation of calcium homeostasis can induce tumor vascular collapse. We summarize recent advances in calcium-based nanoparticles, transarterial embolization strategies, and combination therapies, discuss the molecular mechanisms underlying calcium-mediated endothelial dysfunction, thrombosis, and vascular disruption, compare these approaches with conventional vascular-targeting therapies, and highlight the major challenges and future opportunities for clinical translation.

Terminology clarification: Throughout this review, the term “vascular collapse” is mentioned as a prominent advantageous effect of nanoparticles in treating tumor tissues. It refers to the compromised perfusion in tumor tissues due to their incapability to maintain its vascular network [10,11]. In addition, terms with similar meaning to vascular collapse are also stated in this literature, including vascular disruption, vascular shutdown, vascular occlusion, and vessel obstruction. Although they are distinct terms by definition, they all refer to events that eventually progress to complete collapse of the vascular system in tumor cells. Precisely, vascular disruption is defined as injury of the endothelial tissues that partly compromises circulation, [12,13,14,15], whereas vascular shutdown refers to complete cessation of blood flow [13,14,15]. Physiologically vascular shutdown follows vascular disruption and ultimately leads to ischemia and necrosis of tumor tissues [13,14,15]. Vascular occlusion is a condition of blocked tumor vessels due to coagulation, thrombosis, embolization or calcium-mediated intravascular mineralization [16,17]. These pathologies also compromise tumor perfusion [16,17]. In this review, these terminologies are stated in the context of their mechanisms described in referenced articles.

## 2. Calcium Signaling in Vascular Physiology and Tumor Vasculature

### 2.1. Physiological Role of Calcium in Vascular Cells

The integrity of normal vascular systems is maintained through a highly complex network in its physiological structure [18]. This structure is composed of endothelial cells (ECs), vascular smooth muscle cells (VSMCs), pericytes, and an intact basement membrane [18]. Normal vasculature is essential for regulating tissue perfusion to ensure efficient oxygen and nutrient transfer throughout the tissue [18]. The intracellular calcium signaling enables the vascular cells to communicate. This is necessary for adapting to any physiological stimuli [19]. In case of induced calcium dyshomeostasis, as caused by calcium-based nanoplatforms, the cell signaling is impaired, which in turn impairs vascular function [19]. If the condition remains unchecked and unstabilized, it leads to several pathological alterations as observed in many vascular diseases [19].

Calcium ions (Ca^2+^) serve as versatile and tightly regulated second messengers in vascular physiology, orchestrating a complex cross-talk between endothelial cells (ECs) and vascular smooth muscle cells [20]. Ca^2+^ signaling regulates vascular permeability and nitric oxide generation in ECs, whereas it modifies blood pressure and vascular tone in VSMCs [19]. Several channels, pumps, and exchangers work together to maintain Ca^2+^ homeostasis [21]. Misregulation of this signaling pathway leads to several cardiovascular pathologies such as arterial hypertension, atherosclerosis, and vascular calcification [19]. The most outstanding aspect is that Ca^2+^ signaling by vascular cells is regulated by sex, with the female VSMCs expressing less cytosolic Ca^2+^ than their male counterparts [21]. Estrogens and androgens affect Ca^2^+-handling proteins through genomic and non-genomic mechanisms, contributing to sex-specific differences in vascular function and disease susceptibility [21].

Simultaneously, calcium plays a crucial role in the regulation of vascular function by several pathways. In ECs, Ca^2+^ signaling modulates paracellular permeability by controlling the assembly and disassembly of intercellular junctions, including tight as well as adherens junctions [22]. Influx of Ca^2+^ in vascular smooth muscle cells initiates contraction by activating myosin light chain kinase (MLCK) through a pathway that includes binding to calmodulin resulting in phosphorylation of myosin enabling it to participate in contractile activity [23]. MLCK also plays some role in the flux regulation of Ca^2+^ in smooth muscle cells, potentially influencing Ca^2+^ channel trafficking [24]. Ca^2+^ signaling in smooth muscle cells also opens up pathways for excitation–transcription coupling interacting with post-transcriptional control mediated by microRNAs [25]. All these processes dependent on calcium regulate vascular tone, blood pressure, and endothelial barrier function; their dysregulation is most often associated with vascular diseases.

Furthermore, calcium signaling is integral to angiogenesis and vasculogenesis; it governs the endothelial cell proliferation, migration, and tube formation [26,27]. Intracellular Ca^2+^ concentration is regulated by several mechanisms, including release from intracellular stores and entry through plasma membrane channels [19]. VEGF and FGF, pro-angiogenic factors, trigger Ca^2+^ signals via two pathways: inositol-1,4,5-trisphosphate receptors and store-operated Ca^2+^ entry represented by Orai1 channels [26]. Additionally, lysosomal Ca^2+^ release via two-pore channels constitutes a significant pathway for promoting angiogenesis [26]. Ca^2+^ signaling misregulation results in various vascular pathologies, such as hypertension and atherosclerosis, as well as vascular calcification [19]. Understanding the Ca^2+^ signaling molecular mechanisms in endothelial cells could lead to new therapeutic strategies for targeting angiogenesis in cancer and other disorders [28].

### 2.2. Tumor Vasculature: A Fragile Equilibrium

Unlike the orderly and hierarchically organized vessels of healthy tissue, tumor-associated vasculature exists in a state of dynamic instability and functional chaos, characterized by irregular shapes, tortuosity, and lack of hierarchical organization [29]. These vessels are very dynamic, undergoing constant remodeling and instability [30]. The TME is marked by hypoxia, acidosis, and high interstitial pressure, which contribute to the tumor vasculature aberrant nature [31]. Poor medication distribution, heterogeneous blood flow, and decreased treatment efficacy are the combination of these factors [32]. Both endothelial cells and pericytes in tumors are abnormal, leading to defective basement membrane formation and increased vascular permeability [28]. The combination of these factors results in heterogeneous blood flow, impaired drug delivery, and reduced treatment efficacy [33]. Comprehending these vascular anomalies is essential for creating tailored treatments and enhancing the results of cancer treatment.

**Figure 1:**
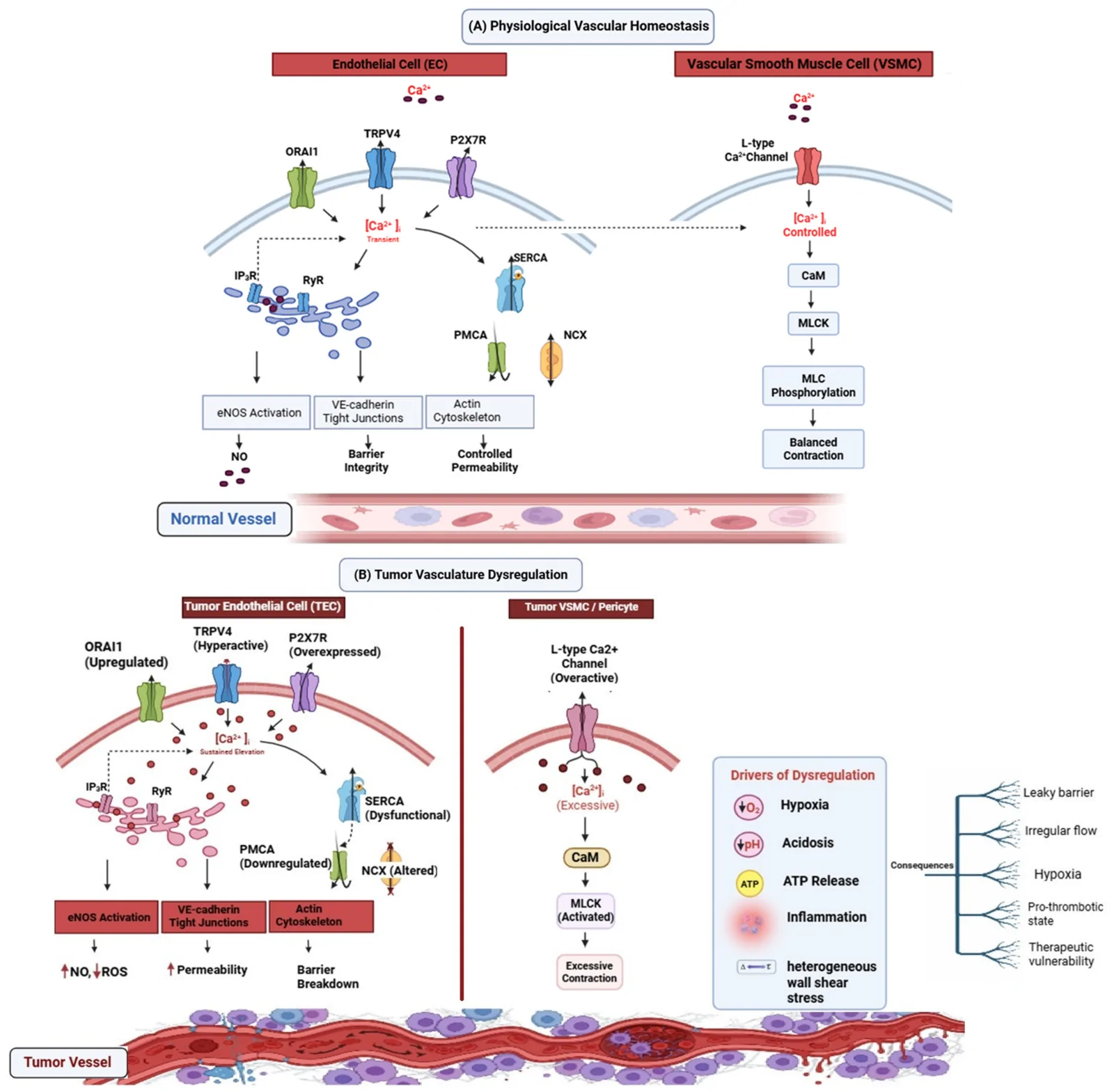
The calcium vulnerability axis: From physiological signaling to tumor vascular fragility.

The distinct features and possible vulnerabilities of tumor endothelial cells (TECs) are attributed to their altered calcium signaling in comparison to normal endothelial cells [7,34]. The calcium toolkit remodeling in TECs involves changes in expression and function of various channels, including TRP, Orai, and P2X7 receptors [35]. TRP, Orai, and P2X7 receptors are among the channels whose expression and function are altered as a result of the calcium toolbox remodeling in TECs (Figure 1) [9]. Pro-angiogenic growth factors elicit intracellular increases in calcium, which transduce specific information for cellular processes under both physiological and pathological conditions [9]. The resistance of TECs to anticancer therapies is explained by a dysregulation in calcium signaling, this makes it a potential target for new therapeutic approaches [35]. Comprehending the intricate relationship between calcium signaling and tumor vascularization could result in more effective strategies development impedes the growth and dissemination of tumors [7].

## 3. Calcium-Induced Vascular Collapse: Mechanisms and Pathways

### 3.1. Solidification via Calcium-Triggered Coagulation

A major pathway by which calcium induces vascular collapse is mediated through the fast induction of localized coagulation and thrombotic solidification within tumor blood vessels. Calcium-based nanotechnology provides potential strategies for cancer therapy by targeting tumor vasculature and exploiting the role of calcium in cellular signaling [36]. The extensively studied platforms including calcium phosphate (CaP), calcium carbonate (CaCO_3_), and calcium peroxide (CaO_2_) accumulate in the microacidic environment of abnormally structured tumors [37,38]. Here, they release Ca^2+^ ions that disrupt calcium homeostasis. This process leads to mitochondrial dysfunction, excessive ROS generation, oxidative stress, and apoptotic cell death, which destabilizes the endothelial barrier and promotes vascular collapse [37,38,39,40]. Smart nanotherapeutics can selectively disrupt tumor blood vessels, inducing thrombosis and vascular infarction to starve tumors [41]. These tumor vascular disrupting agents (Tumor-VDAs) take advantage of the unique characteristics of tumor vasculature, offering an alternative approach to standard therapies [42]. Combining tumor-infarction therapy with chemotherapy, for instance, co-delivering thrombin and doxorubicin in tumor-targeted nanoparticles, has proved to enhance tumor growth suppression and reduce recurrency in animal models [43]. Calcium-based nanotechnology can interfere with calcium homeostasis to activate immune cells or induce tumor cell death by different mechanisms, including cellular calcium overload and disrupted intracellular communication [36]. These novel approaches represent promising strategies toward improvement of cancer treatment outcomes.

Recent research has examined new methods of targeting tumor vasculature and causing tumor cell death. Calcium overload-based ion interference therapy has emerged as a promising strategy to overcome resistance to conventional treatments [44]. This method involves the rapid accumulation of calcium ions in the tumor tissue, resulting in mitochondrial dysfunction, reactive oxygen species (ROS) production, and cell death (necrotic or apoptotic) [45,46]. The endothelial injury, in turn, promotes platelet activation, localized thrombosis and progressive vascular occlusion. In the process, they create a self-amplifying cycle that deprives tumors of oxygen and nutrients [3]. Similar to calcium-based therapeutics, the calcium/copper peroxide nanocomposites enhance vascular disruption by simultaneously inducing Ca^2+^ overload and generating hydroxyl radicals through Cu^2+^-mediated Fenton-like reactions [47]. The metabolic waste produced in the form of H_2_O_2_ is reused during the reaction that amplifies oxidative stress, mitochondrial injury, and tumor calcification [47]. Irreversible vascular and tissue damage in tumors is also achieved with bioresponsive calcium-derived nano-assemblies that follow the same mechanism of calcium dyshomeostasis and the associated damage [46]. Another effective strategy is the co-delivery of thrombin and doxorubicin, which selectively bind fibrin–fibronectin complexes within tumor vessels to trigger localized coagulation and vascular infarction [43]. The simultaneous delivery of chemotherapeutic agents produces synergistic antitumor effects and reduces tumor recurrence in preclinical models [43]. Collectively, these approaches exhibit potent strategies of starving tumors of their blood supply.

### 3.2. Endothelial Cell Apoptosis and Collapse

Other than coagulation-driven vessel obstruction, elevated intracellular calcium levels can lead to the apoptosis of endothelial cells and the tumor vasculature structural collapse [48,49]. This mechanism entails a calcium-induced cascade of intracellular events that culminate in programmed cell death, different from necrosis and with significant implications for vascular integrity [50,51,52]. Increased calcium influx through store-operated calcium entry (SOCE) channels like Orai1 and TRPC1 further exacerbates cellular imbalance in tumor endothelial cells, which are already under metabolic stress from oxidative stress, lack of nutrients, and hypoxia [53,54,55,56]. Once calcium in the cytosol exceeds a physiological threshold, it disrupts mitochondrial membrane potential (Δψm) [57,58], activates calcium-dependent proteases (e.g., calpains), and initiates the release of cytochrome c, hallmarks of intrinsic apoptosis [59,60,61,62].

Crucially, calcium-induced apoptosis is not solely a passive consequence of the overloading of ions, but a programmable mechanism that can be exploited therapeutically [38,41,50]. The calcium ionophores targeted delivery, calcium-mimetic nanoparticles, or agents that disrupt calcium homeostasis in a specific manner in tumor vasculature has shown the potential to trigger endothelial failure without affecting adjacent normal vessels [47,63,64,65,66,67,68]. This spatial selectivity is attributed to the intrinsic instability of tumor vessels and their exaggerated sensitivity to ionic perturbations, a vulnerability that can be exploited to selectively prune the vascular network feeding solid tumors [69,70,71,72,73]. The outcome is a rapid, irreversible tumor mass devascularization, which causes nutrient deprivation, ischemia, and secondary tumor cell death [69,71,72].

### 3.3. Hierarchical Vascular Shutdown in Tumor Vasculature

Tumor vasculature is organized hierarchically, with upstream feeding vessels supporting descending capillary networks. Calcium-based agents can be designed to selectively target these feeder vessels, thereby initiating a cascading collapse of vasculature that isolates tumors from systemic perfusion. Whereas embolic agents and their mechanisms are well documented, the hierarchical vascular shutdown specific process has not been thoroughly described in the literature [74]. Unlike the extensively studied inhibition of angiogenic signaling pathways such as VEGF, the sequential structural events that progress from localized vessel occlusion to the collapse of broader vascular hierarchies remain poorly understood [74]. Clarifying these mechanisms is crucial, as such knowledge could guide the targeted interventions and standardized protocols development for tumor vascular management.

The vascular shutdown downstream effects are largely driven by tumor hypoxia, which completely remodels the tumor microenvironment. Hypoxia causes the hypoxia-inducible factors (HIFs) release, which in turn promote invasion, metastasis, and the epithelial–mesenchymal transition (EMT) [75,76]. Additionally, it creates an immunosuppressive milieu that restricts anticancer immune responses and contributes to poor prognosis [75]. Tumors then adjust to ischemic stress through mechanisms such as vessel co-option and vascular mimicry, which allow them to exploit host vasculature or form non-endothelial conduits for nutrient supply [76]. These adaptations undermine the efficacy of vascular-targeted therapies and promote resistance. Furthermore, hypoxic regions recruit tumor-associated macrophages (TAMs) and other immune cells that secrete pro-tumorigenic factors, which aids in immune evasion and the spread of metastases [77]. Together, these processes demonstrate the dual challenge of addressing the tumor blood supply while mitigating hypoxia-driven adaptations and resistance mechanisms.

Calcium-based nanomaterials, in particular the calcium phosphate (CaP) nanoparticles, are potential agents for inducing hierarchical vascular shutdown. They have biocompatibility and degradability characteristics in addition to increased permeability and retention (EPR) impact, enabling preferential accumulation within tumor vasculature [78,79]. Once localized, they destabilize the homeostasis of calcium and signaling pathways essential for endothelial survival and vessel stability, leading to selective vascular occlusion or apoptosis of tumor-feeding vessels [80]. This therapeutic effect arises from three mechanisms integration: nanoparticle-mediated delivery, exploitation of tumor microenvironmental features, and targeted disruption of calcium signaling. Optimizing these agents for improved selectivity, delivery efficiency, and reduced systemic toxicity remains a priority, as doing so could establish calcium-based therapies as effective and safe strategies for hierarchical vascular shutdown in clinical oncology.

## 4. Therapeutic Platforms and Delivery Systems

### 4.1. Nanoparticle-Mediated Calcium Delivery

Recent advances in nanotechnology highlights the potential of calcium-based nanomaterials as drug delivery systems to enable sustained disruption of intracellular calcium equilibrium that acts as a cytotoxic trigger for mechanisms such as apoptosis, calcicoptosis (calcium-dependent cell death pathways) and mitochondrial dysfunction [39]. Along with the benefits of nanotherapy, calcicoptosis has emerged as a physiological process of induced cell death due to calcium overload. It is characterized by sustained intracellular Ca^2+^ accumulation that disrupts calcium homeostasis and promotes mitochondrial dysfunction, generation of excessive ROS and bioenergetic collapse [81,82]. These events collectively lead to irreversible cell death [81]. The molecular features of calcicoptosis are similar to apoptosis, necrosis and other regulated cell death pathways [82]. However, they differ in terms of mechanism [82]. While apoptosis and necrosis are mediated by caspase activation and cellular damage, respectively, calcicoptosis is induced by persistent Ca^2+^ overload (dyshomeostasis) that triggers mitochondrial permeability transition, energetic failure and oxidative stress [83]. It is also different from pyroptosis, which activates inflammasomes and induces gasdermin-mediated pore formation in membranes [84,85].

**Figure 2:**
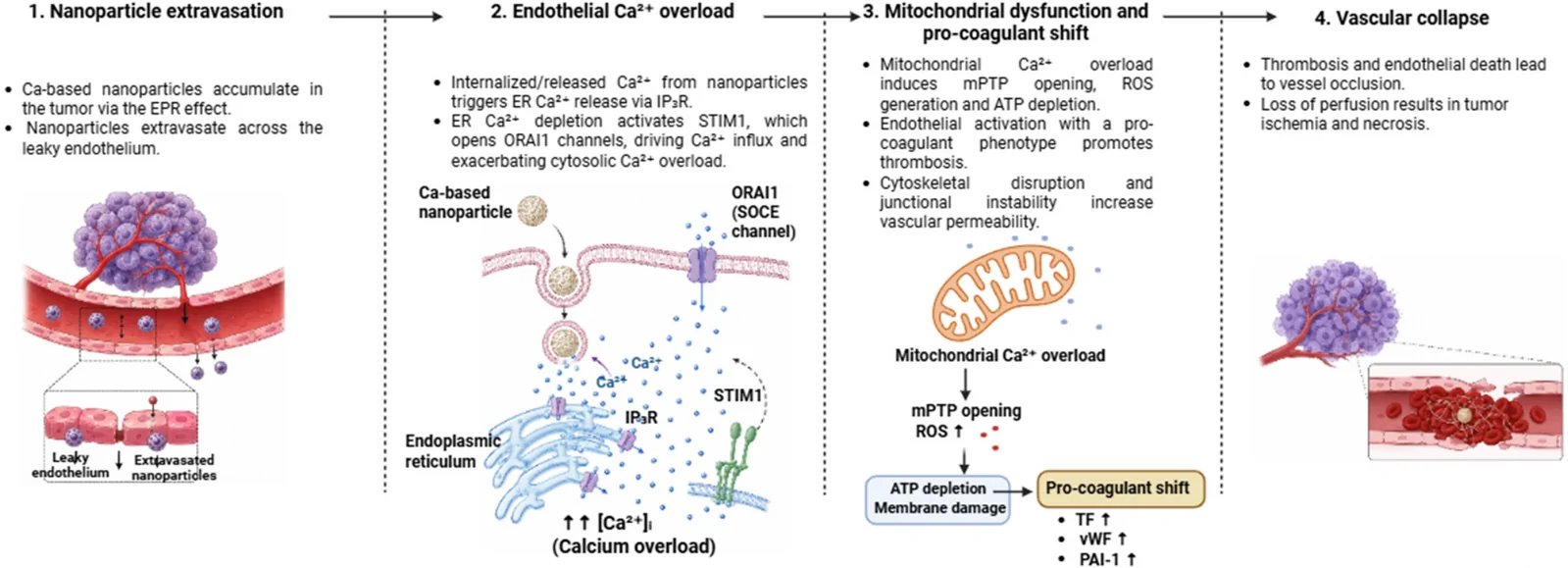
Cellular and molecular mechanisms underlying calcium-induced tumor vascular collapse.

This approach is precise and uses active biocompatible therapeutic agents in the form of calcium phosphate (CaP), calcium carbonate (CaCO_3_), and calcium peroxide (CaO_2_), instead of passive carriers commonly used in conventional drug delivery systems (Table 1). Precisely, following cellular internalization, the above nanomaterials undergo controlled degradation in tumor microenvironments, such as acidic pH and oxidative stress. This process releases calcium ions and induces intracellular calcium overload [86,87,88]. The resulting calcium surge overwhelms the cellular buffering systems, and promotes mitochondrial Ca^2+^ uptake, which compromises mitochondrial membrane potential and ATP generation, and results excessive reactive oxygen species (ROS) accumulation. Altogether these events reinforce oxidative-stress-mediated cytotoxicity and activate multiple programmed cell death pathways (Table 1) [81,82,87,89,90]. Following the process of degradation of nanoplatforms, the calcium surge overwhelms the cellular buffering systems. This event promotes opening of the mitochondrial permeability transition pore (mPTP) and results in mitochondrial membrane depolarization, matrix swelling, ATP depletion, and excessive ROS production [58,91,92]. In addition, the calcium surge activates calcium-dependent proteases (such as calpains), which cleave cytoskeletal and mitochondrial proteins, and thereby amplify cellular damage [93]. The prolonged opening of mPTP leads to outer mitochondrial membrane rupture, release of pro-death mediators, and irreversible mitochondrial dysfunction (Figure 2) [94,95]. Also, cells can undergo apoptotic or necrotic death on prolonged calcium overload [96]. Overall, calcicoptosis is an upstream calcium-driven mechanism that can converge with diverse regulated cell death pathways [97].

In the endothelial cells, calcium overload disrupts actin cytoskeleton organization and weakens adherens junctions. As a result, vascular permeability increases and endothelial contraction is induced, ultimately leading to structural collapse of tumor vessels [98]. The vascular integrity is further compromised due to activation of coagulation cascade and fibrin deposition which leads to thrombosis, restricting tumor blood supply [99,100]. In the endoplasmic reticulum (ER), which serves as the primary regulator of intracellular Ca^2+^ storage and signaling, calcium overload overwhelms Sarco/Endoplasmic Reticulum Ca^2+^-ATPase (SERCA) and ion channels such as inositol 1,4,5-trisphosphate receptors (IP_3_Rs) and ryanodine receptors (RyRs), and impairs store-operated calcium entry (SOCE). During this event, SERCA, IP_3_Rs and RyRs exacerbate Ca^2+^ release from ER stores while SOCE prevents restoration of equilibrium (Figure 2). The disrupted equilibrium induces ER stress, protein misfolding, and activation of unfolded protein response pathways, and further converges with mitochondrial-mediated and caspase-dependent apoptosis [81,101,102].

**Table 1:** Calcium-based therapeutic platforms in cancer therapy.

Platform	Main Material	Typical Particle Size (nm)	Drug/ Functional Cargo	Ca^2+^ Release Characteristics	Primary Mechanism of Action	Representative in Vivo Antitumor Efficacy *	References
**Calcium Phosphate Nanoparticles**	CaP	80–150	Doxorubicin, genes, imaging agents	Acid-responsive dissolution in the tumor microenvironment	Intracellular Ca^2+^ overload, mitochondrial dysfunction, endothelial apoptosis	>70% tumor inhibition in murine models; reduced systemic toxicity	[86,103]
**Calcium Carbonate Nanoplatforms**	CaCO_3_	100–200	Sonosensitizers, photosensitizers, chemotherapeutics	Rapid pH-triggered Ca^2+^ release	Calcium overload, ROS amplification, mitochondrial damage	Up to **90.9% tumor inhibition** and **80% 60-day survival** in SDT-treated mice	[88,89]
**Calcium Peroxide Systems**	CaO_2_	100–200	Photosensitizers or therapeutic agents	pH-triggered release of Ca^2+^ and O_2_	Oxygen generation, ROS amplification, calcium overload	Significant suppression of tumor growth in PDT/PTT preclinical models	[87,104]
**Lipid-Coated CaP Nanocarriers**	CaP + lipid shell	80–120	Doxorubicin	Controlled pH-responsive co-release of DOX and Ca^2+^	Combined chemotherapy and calcium overload	Enhanced tumor accumulation with reduced systemic toxicity compared with free DOX	[103]
**Calcium Embolization Systems**	Calcium-based embolic agents	Micrometer-sized embolic particles	None	Sustained local Ca^2+^ availability within occluded vessels	Coagulation activation, fibrin formation and thrombosis	Efficient vascular occlusion and tumor infarction in embolization models	[100]
**Hybrid Calcium Nanotherapies**	Multifunctional calcium nanoplatforms	50–200	Chemotherapy, PDT, SDT, immunotherapy agents	Stimuli-responsive programmable Ca^2+^ release	Synergistic multimodal therapy	Superior antitumor efficacy compared with corresponding monotherapies	[105,106]

* Representative in vivo antitumor efficacy reported in the cited preclinical studies.

The preferential accumulation of nanomaterials within tumor tissues is described as the enhanced permeability and retention (EPR) effect, and it arises due to aberrant vascular architecture and deficient lymphatic clearance systems [39,88]. A recent study further indicated that the EPR effect can be further exploited to modulate tumor vascular structures with nitric-oxide-mediated basement membrane permeabilization to further enhance nanoparticle penetration and distribution within tumor tissues [107]. To further improve selectivity and minimize off-target toxicity, the nanocarrier-mediated drug delivery systems are improvised through functionalization with tumor specific receptors, such as peptides, antibodies, interleukins (for instance, IL-13 which selectively binds IL-13Rα2 receptors overexpressed in glioma cells) or biomimetic coatings [108].

The biological differences in tumor and normal cells favor the enhanced permeability and retention (EPR) effect of calcium-based nanoplatforms for selective targeting. Studies have emphasized that although the EPR effect is pronounced and reproducible in rapidly growing murine xenograft models, they are generally less effective in human tumors [109]. In the former example, the immature and highly permeable vasculature along with poor lymphatic drainage in murine xenograft allows retaining of high dose therapeutics at the target site [110]. In contrast, since human tumors are heterogeneous in terms of vascular permeability, extracellular matrix organization, stromal density, and interstitial fluid pressure, they substantially limit nanoparticle extravasation and intra-tumor penetration [110,111]. Precisely, although preclinical studies demonstrate accumulation of 5–10% of the injected therapeutic dose, the clinical studies typically achieve accumulation of only approximately 0.7% of the injected dose. This example portrays the limitations of translation of preclinical models in patient therapeutics, and supports the use of preclinical models as proof of concept, and not as a predictor of therapeutic efficiency [112,113]. Thereby, the nanotherapeutic framework should emphasize on active targets and optimum nanoparticle dose that are validated in clinically relevant models and early-phase clinical trials [112,113].

### 4.2. Image-Guided Transarterial Calcium Embolization

The image-guided transarterial embolization (TAE) approach is an increasingly adopted intervention in treatment of hypervascular tumors, particularly hepatocellular carcinoma (HCC). HCC is characterized by tumor growth which is highly dependent on arterial blood supply. Hence, this approach is focused on depriving tumor tissue of oxygen and nutrients through delivery of embolic agents and selective catheterization of tumor-feeding arteries, with the help of real-time imaging guidance. In the process, vessel occlusion and coagulative necrosis is achieved by inducing rapid ischemia [114,115]. Calcium-based embolic systems release Ca^2+^ ions into the tumor vessels which accelerate prothrombin to thrombin conversion to produce more stable and sustained vascular occlusion through fibrin networks, compared to mechanical obstruction of microvasculature achieved by inert embolic agents used in conventional systems [100]. In addition to obstruction, these events alter cytoskeletal organization, weaken intercellular junctions, enhance ischemic conditions within the tumor mass, propagate microvascular thrombosis and amplify vascular shutdown. Thus, under sustained exposure, this approach causes irreversible vessel damage in tumor cells resulting in definitive treatment outcome [82,98].

In terms of mechanism of action, calcium-based embolization is similar to transarterial chemoembolization (TACE). However, instead of cytotoxic drugs used in TACE, the former approach exploits ion-mediated signaling disruption mechanisms. As described in the above section, disruption of calcium homeostasis triggers ER, endothelial and mitochondrial dysfunction, oxidative stress and activates calcium-dependent cell death pathways. Also, compared to TACE, the drug-independent mechanism is particularly useful in treating chemo-resistant tumors [39,81]. Imaging modalities including computed tomography (CT), magnetic resonance imaging (MRI), and digital subtraction angiography (DSA) are central to the success of TAE, since they guide both delivery and evaluation by enabling high resolution visualization of tumor vasculature. These modalities allow selective catheterization and minimize damage to surrounding healthy tissue. Among the various imaging techniques, DSA is commonly preferred due to its real-time assessment of blood flow and embolic distribution [116].

The most practical improvisation of TAE approach is the development of platforms with integrated imaging and therapeutic functionalities. A notable example is lipid-coated calcium phosphate nanoparticles incorporating carbon dots. These systems combine pH-responsive drug release mechanisms with intrinsic imaging capability. They maintain structural stability during circulation but undergo rapid degradation in acidic tumor environments. The pH-responsive drug release mechanism in these systems allows controlled release of encapsulated agents (Ca^2+^ ions or drugs) in tumors through the EPR effect, while the imaging elements allow real-time tracking of nanoparticle distribution [103]. Further improvisation of this strategy includes calcium-based thermosensitizers that have demonstrated the ability to induce persistent cavitation effects and improve energy deposition during ablative therapies [99].

### 4.3. Hybrid Therapies

Hybrid therapeutic platforms integrate calcium-mediated ion dysregulation with additional treatment modalities such as chemotherapy, phototherapy, sonodynamic therapy, or immunomodulation. This approach creates converging stress signals that overcomes compensatory survival pathways of tumor cells, reduces therapeutic resistance, and addresses microenvironmental barriers of hypoxia and limited drug penetration [39,105,106]. Among these methods, the use of engineered calcium-based nanocarriers with encapsulated doxorubicin (chemotherapeutic agents) is widely used as a co-delivery system. This system allows improved efficacy through amplified apoptotic signaling and reduced threshold for drug-induced cytotoxicity, achieved by simultaneous Ca^2+^-overload-mediated homeostasis disruption and chemotherapeutic-agent-induced inhibition of DNA replication and cell cycle progression [103,108].

In photodynamic therapy (PDT) integrated with calcium peroxide (CaO_2_)-based nanoplatforms, oxygen is released in tumor cells along with Ca^2+^ ions to induce and sustain ROS production and alleviate hypoxia during light irradiation [87,117]. This enhances PDT efficiency by establishing a feedback loop in which ROS damages calcium regulating organelles, intensifying intracellular Ca^2+^ accumulation [86,88]. In photothermal therapy (PTT), light energy is converted into localized heat which directly disrupts cellular structures and enhances ion flux across membranes. Integration of PTT with calcium-based nanomaterials hyperthermia accelerates nanoparticle degradation, promotes rapid Ca^2+^ influx and induces ferroptosis. Altogether, this action creates a highly hostile intracellular environment in tumors [90].

Ultrasound-based modalities such as sonodynamic therapy (SDT) is helpful in treating deep tissue tumors where light penetration is limited. A recent pH-responsive mesoporous CaCO_3_ nanoplatform combined with SDT demonstrated quantitative therapeutic outcomes, including a 71% loss of mitochondrial membrane potential, a 1.6-fold increase in intracellular ROS generation, 94.2% calreticulin exposure, 46.2% HMGB1 release, 74.5% ATP depletion, 90.9% tumor inhibition, and an 80% 60-day survival rate in treated animals [89]. These findings support the role of a Ca^2+^–ROS positive feedback loop in amplifying sonodynamic therapy. Another interesting hybrid system combines calcium overload with ion-mediated or redox-based therapies. For example, hydrogen-releasing systems or metal-ion-based therapeutics modulate intracellular redox balance. Integrating these systems with calcium-based nanoparticles leads to the convergence of multiple ion fluxes that destabilize cellular homeostasis, and activates antitumor immune responses [118,119].

More recent nanoplatforms incorporate immunotherapeutic components to exploit calcium-induced immune activation. Besides calcium overload and ROS bursts, these systems demonstrate enhanced immunogenic cell death (ICD) through calreticulin exposure, ATP release, and High-Mobility Group Box 1 (HMGB1) secretion. For example, the SDT-based calcium carbonate platform reported 94.2% calreticulin exposure, 46.2% HMGB1 release, and marked ATP depletion, indicating robust ICD induction [89]. Collectively, they promote dendritic cell maturation and activation of cytotoxic T lymphocytes, leading to the release of tumor-associated antigens and damage associated molecular patterns (DAMPs) [106,120]. Similarly, approaches such as cGAS–STING activation or thrombosis-induced immune modulation simultaneously target vascular and immune effects to achieve durable antitumor responses [104,106].

Overall, the nanoparticle-mediated calcium delivery systems, including hybrid platforms, ensure a promising approach in cancer treatment through multiple modes of actions that enhances therapeutic efficacy and reduces the likelihood of resistance development.

## 5. Preclinical and Clinical Insights

### 5.1. Preclinical Evidence

Preclinical investigations have consistently indicated localized ionic imbalance through EPR effect and vascular disruption induced by calcium-based nanomaterials [81,88,102]. Increased vascular permeability and structural destabilization have been successfully demonstrated in in vivo studies in murine tumor models that revealed disrupted cytoskeletal organization and weakened intercellular junctions on sustained elevated levels of Ca^2+^ in tumor endothelial cells [98,99]. Wang et al. [37] reported that calcium-based nanoplatforms were capable of triggering intra-tumoral thrombosis and vascular infarction, leading to significant tumor growth inhibition in mouse models.

Besides monotherapy, combinational strategies integrating calcium-mediated therapies with other approaches have shown strong potential in enhancing treatment efficacy. For instance, Zeng et al. [99] reported that calcium-carbonate-based thermo-sensitizing nanoplatforms significantly improve radiofrequency ablation (RFA) outcomes in HCC models. The study indicated that these systems promote cavitation effects, and improve thermal energy transfer within tumor tissues by releasing Ca^2+^ and carbon dioxide under acidic conditions. This mechanism is further reported to be superior to conventional ablation approaches, since RFA leads to more uniform heat distribution and enhanced vascular disruption in tumor cells. Similarly, improved tumor suppression has been reported through the use of calcium-phosphate-based composites that simultaneously cause localized heating and calcium-induced apoptosis [121].

The preclinical evidence has further shown the efficacy of Ca^2+^–ROS positive feedback loop in PDT and SDT that intensifies oxidative stress on use of calcium-peroxide-based systems [87,117] and CaCO_3_ nanoplatforms [89], respectively, in in vivo models. Chen et al. [89] developed a hyaluronic-acid-modified mesoporous calcium carbonate nanoplatform and combined it with SDT for treating deep seated solid tumors. The study demonstrated a 71% collapse in membrane potential and a 1.6-fold increase in ROS generation. Additionally, the platform targeted CD44 cells and triggered ICD, enhancing calreticulin exposure by 94.2%, release of HMGB1 by 46.2%, and ATP decrease by 74.5%. Overall, 90.9% tumor inhibition and 80% 60-day survival rate was achieved. Among more emerging evidence, Wang et al. [104] developed a core–shell nanoplatform comprising kaempferol-loaded calcium peroxide nanoparticles coated with a PEG tannic acid/manganese metal phenolic network. This system induced severe redox imbalance via concomitant GSH depletion and ROS burst. Moreover, they reported release of DAMPs (including calreticulin and HMGB1) in breast cancer cells due to tannic acid enhanced membrane affinity, which triggered ICD.

The most effective preclinical efficacy has been reported with integration of calcium-based nanomaterials and chemotherapeutic agents. Zhang et al. [103] used lipid-coated calcium phosphate nanoparticles co-loaded with doxorubicin in in vivo models and demonstrated its superior tumor accumulation and reduced systemic toxicity compared to free drug formulations. The study further incorporated carbon dots within the nano-systems to allow real-time tracking of nanoparticle distribution and therapeutic response. Through this theranostic platform, the study indicated that the dual-release system enhances cellular uptake, facilitates endosomal escape, and increases cytoplasmic drug availability. Overall, the preclinical evidence supports calcium-mediated vascular disruption as an efficient anticancer strategy.

### 5.2. Early Clinical Observations

Presently, most of the reported evidence of efficacy of calcium-based nanotherapeutics is confined to preclinical models. The clinical translation remains at an early stage. There are no large-scale clinical trials demonstrating the efficacy of calcium-induced vascular disruption or calcium-overload-mediated tumor ablation in human patients. Well-designed clinical studies are required to establish safety, optimal dosing, pharmacokinetics, and therapeutic efficacy in humans. Additionally, such studies are necessary to characterize patient specific factors such as tumor type, vascular architecture and microenvironmental conditions. While localized delivery via nanoparticles or intra-arterial administration is designed to minimize off-target effects, the long-term safety of sustained or repeated calcium release in vivo remains insufficiently characterized. In particular, risks associated with ectopic calcification, unintended thrombosis, and disruption of normal vascular function requires careful evaluation in clinically relevant models [103,108].

Many studies on calcium phosphate and related nanoparticles have focused on their biocompatibility, biodegradability, and drug delivery potential [86,88]. Similarly, nanoplatforms such as lipid-coated calcium phosphate systems have been investigated in translational or clinically oriented research and have provided proof of concept for gene delivery and imaging applications [39,102]. These insights will be favorable in supporting future clinical translations.

Overall, despite substantial preclinical evidence, well-designed early-phase clinical trials are currently lacking, and are essential to evaluate safety, establish dosing strategies, and determine therapeutic windows.

### 5.3. Translational Challenges

The basic translational challenge in using calcium-based nanomaterials arises due to the focus of most investigations on small animal models. These models do not fully capture the heterogeneity of human tumors, particularly with respect to vascular architecture, stromal composition, and immune interactions [86]. Consequently, the promising therapeutic outcomes observed in preclinical studies should be interpreted with caution, as their direct extrapolation to human cancers remains uncertain. Tumor heterogeneity further complicates translational outcomes. Tumor microenvironments in different individuals, as well tumors occurring in different locations in same individuals, vary in their pH levels, redox status, vascular density, and stromal composition. This characteristic can significantly influence nanoparticle accumulation in humans, their degradation kinetics, and calcium release profiles. One important manifestation of this biological heterogeneity is the marked variability in extracellular tumor pH (pHe), a key determinant of the behavior of pH-responsive calcium nanoplatforms [122,123,124,125,126]. Murine xenograft models generally exhibit a relatively homogeneous acidic tumor microenvironment, with extracellular pH values commonly around 6.7–6.9, whereas clinical studies have demonstrated substantially greater inter- and intratumoral pH heterogeneity in human solid tumors. Direct measurements in patients reported extracellular tumor pH values ranging from 5.55 to 7.69 (mean 6.81 ± 0.09) and from 5.66 to 7.78 (mean 7.06 ± 0.05), highlighting marked variability among tumor types and even within individual lesions [122,123,124,125,126]. Because many calcium-based nanoplatforms, particularly CaCO_3_ and CaP nanoparticles, rely on acidic conditions to trigger dissolution, this pronounced spatial and temporal pH heterogeneity can markedly alter nanoparticle degradation kinetics, Ca^2+^ release, and ultimately therapeutic efficacy. Consequently, formulations optimized in murine models may exhibit reduced predictability and reproducibility in clinical settings [39,88]. The biological differences in tumor and normal cells favor the enhanced permeability and retention (EPR) effect of calcium-based nanoplatforms for selective targeting. Studies have emphasized that although the EPR effect is pronounced and reproducible in rapidly growing murine xenograft models, they are generally less effective in human tumors [109]. In the former example, the immature and highly permeable vasculature along with poor lymphatic drainage in murine xenograft allows retaining of high dose therapeutics at the target site [109]. In contrast, since human tumors are heterogeneous in terms of vascular permeability, extracellular matrix organization, stromal density, and interstitial fluid pressure, they substantially limit nanoparticle extravasation and intra-tumor penetration [110,111]. Precisely, although preclinical studies demonstrate accumulation of 5–10% of the injected therapeutic dose, the clinical studies typically achieve accumulation of only approximately 0.7% of the injected dose. This example portrays the limitations of translation of preclinical models in patient therapeutics, and supports the use of preclinical models as proof of concept, and not as a predictor of therapeutic efficiency [112,113]. Thereby, the nanotherapeutic framework should be assessed on active targets and optimum nanoparticle doses that are validated in clinically relevant models and early-phase clinical trials [112,113].

This limited accumulation may reduce the local calcium concentration below the threshold required to induce effective endothelial calcium overload and vascular shutdown, thereby diminishing therapeutic efficacy. Another issue lies in the engineering of multifunctional nanoplatforms that can maintain structural stability in circulation while enabling precise, stimulus-responsive release of Ca^2+^ and co-delivered therapeutic agents within tumor tissue. Achieving this level of spatiotemporal control is particularly demanding, as premature degradation may result in off-target calcium release, whereas insufficient responsiveness can limit therapeutic efficacy [86,88].

Among the study designs, variations in nanoparticle size, composition and surface functionalization complicate cross-study comparisons and hinder standardization. Additionally, information on key factors such as long-term biosafety, pharmacokinetics, long-term biodistribution, and potential systemic toxicity of calcium-based nanomaterials remains limited, and hence they are not yet fully understood [39,86]. Comprehensive studies addressing clearance pathways, potential accumulation in off-target organs, and interactions with physiological calcium regulation systems are also limited [39,102].

Safety considerations are a critical barrier in clinical implementation of calcium-based therapeutics. Although these materials are generally considered safe and biocompatible, the possibility of dysregulated Ca^2+^ release at non-target sites cannot be overlooked. Such instances can disrupt normal cellular functions, especially in highly active tissues such as cardiac and neural systems. It can also lead to unintentional coagulation or ectopic calcification in vascular compartments. Since many therapeutic strategies intentionally induce calcium overload or thrombosis within tumors, it is necessary to carefully evaluate dose thresholds. Also, suitable strategies to ensure spatial confinement are essential to avoid adverse effects of calcium overdose in normal cells [81,82].

Finally, these combined biological, technological, and safety challenges are reflected in the current stage of clinical development. To date, no calcium-based nanomedicine specifically developed for calcium-overload-mediated tumor vascular disruption has entered Phase I clinical trials. Although calcium phosphate nanoparticles have been investigated as drug and gene delivery platforms [127,128], therapeutic systems designed to induce vascular collapse through calcium overload remain at the proof-of-concept or animal validation stage. Future studies should therefore focus on developing active targeting strategies, optimizing nanoparticle design, validating these systems in clinically relevant large-animal models, and ultimately establishing their safety and efficacy through carefully designed first-in-human clinical trials to facilitate successful clinical translation.

**Figure 3:**
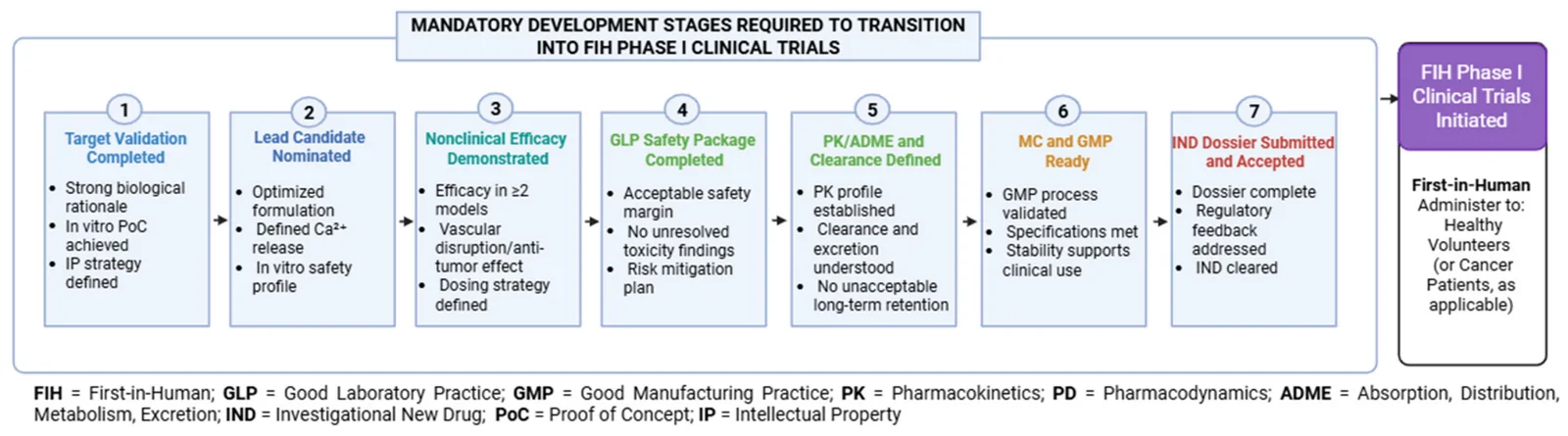
Mandatory developmental stages required for the clinical translation of calcium-based vascular-targeting nanotherapeutics into first-in-human (FIH) Phase I clinical trials.

Overall, although calcium-based nanotherapeutics have demonstrated encouraging preclinical efficacy [37], the available evidence remains largely restricted to small animal models. Consequently, their therapeutic performance in the highly heterogeneous clinical setting of human tumors remains uncertain. Well-designed translational studies, followed by carefully conducted clinical trials, are therefore required to determine whether the promising results observed in experimental models can be successfully translated into meaningful clinical benefit for patients (Figure 3). Successful clinical translation requires target validation, lead candidate optimization, comprehensive nonclinical safety evaluation, Good Laboratory Practice (GLP)-compliant toxicology studies, pharmacokinetic and pharmacodynamic characterization, establishment of Good Manufacturing Practice (GMP)-compliant manufacturing, and regulatory approval through submission of an Investigational New Drug (IND) application [129]. These sequential steps are mandatory to ensure product quality, reproducibility, safety, and regulatory compliance before clinical evaluation (Figure 3).

## 6. Advantages over Traditional Vascular-Targeting Therapies

Calcium-mediated vascular disruption offers several distinct advantages over conventional vascular-targeting strategies (Table 2), particularly vascular disrupting agents (VDAs) and anti-angiogenic therapies. One of the most notable benefits of the former therapy is the rapid and irreversible shutdown of tumor vasculature. The conventional VDAs typically act by destabilizing endothelial cells or inhibiting cytoskeletal integrity. These processes require sustained drug exposure and exhibit delayed therapeutic effects. In contrast, calcium-based approaches induce immediate vascular occlusion through physicochemical processes such as calcium salt precipitation, intravascular aggregation, and embolic blockage. Collectively, these processes lead to acute ischemia and extensive tumor necrosis [105,106]. The irreversible nature of this vascular collapse is particularly advantageous, as it limits the window for tumor adaptation and significantly reduces the likelihood of vascular regrowth or functional recovery [106].

Another important advantage of calcium-induced vascular disruption lies in their underlying solid state physicochemical mechanism. The conventional VDAs depend on biochemical interactions with endothelial cells, which can vary depending on tumor type, microenvironment, and genetic heterogeneity. Hence, VDAs are associated with drawbacks such as inconsistent therapeutic outcomes and limited predictability of treatment responses. In contrast, calcium-based therapies operate through physicochemical mineralization processes, such as mineral deposition and physical luminal blockage. These processes are comparatively less susceptible to biological variability and hence confer greater predictability and consistency in therapeutic outcomes [77].

A major advantage of calcium-mediated strategies is their reduced propensity for development of resistance (Table 2). Besides drug resistance, tumors often develop resistance to anti-angiogenic therapies through alternative angiogenic pathways. However, vascular occlusion mediated by calcium deposits presents a more difficult physical barrier for tumors to overcome, as it does not rely on modulating specific molecular targets [77,133].

In tumors, the abnormal structure of vasculature, characterized by high permeability, irregular architecture, and impaired lymphatic drainage, facilitates the preferential accumulation of calcium-based nanomaterials. The EPR effect enables localized calcium release and targeted vascular disruption, minimizing damage to normal tissues [86,130]. This tumor selectivity can be exploited for achieving more selective therapeutic effects compared to conventional agents that often exhibit systemic toxicity (Table 2) [130].

In addition to the above advantages, calcium-mediated therapies show synergistic potential with other treatment strategies, such as chemotherapy, immunotherapy, and photothermal approaches. Studies have shown that combination therapies can enhance drug retention within the tumor cells and promote vascular shutdown. At the same time, it can also induce and promote ICD and oxidative stress. Thus, calcium-mediated therapeutics can significantly amplify the therapeutic efficacy of immunotherapy and phototherapy [102,131,132].

Lastly, the most desirable advantage of calcium-based systems is their cost effectiveness and accessibility compared to complex biological or targeted agents. This economic advantage favors their broad clinical application, particularly in resource limited settings [39,106].

## 7. Challenges and Future Perspectives

### 7.1. Safety Considerations

A primary concern with the use of calcium-mediated anticancer therapeutics is their potential for uncontrolled or off-target release. Calcium acts as a key molecule in coagulation pathways and intracellular signaling. Calcium overload also triggers apoptosis (calcicoptosis) and oxidative-stress-mediated injury. Hence, off-target exposure due to dysregulated delivery systems can cause thrombosis, embolism, or damage to healthy vascular tissues [81,82,98,101]. As a result, in-depth understanding of calcium dosage, release kinetics, and localization is necessary before the use of these systems is approved in clinical trials.

### 7.2. Enhancing Delivery Systems

The delivery platforms are continuously improvised to achieve precise and controlled calcium release and address safety challenges. A recent progress in this context is represented by stimuli-responsive nanocarriers that exploit tumor microenvironment conditions such as acidic pH, hypoxia, or enzymatic activity. Hence, to a large extent, these models reduce off-target effects and enhance therapeutic selectivity [89,102]. In addition, the biomimetic strategies (such as cell-membrane-coated nanoparticles and protein-based carriers) have gained attention for their ability to evade immune recognition. These systems improve tumor accumulation and enhance delivery efficiency by mimicking natural biological interfaces [107,108]. Furthermore, multifunctional nanoplatforms offering real-time monitoring and synergistic therapeutic effects are being increasingly explored for their treatment efficiency [37,106]. Although remarkable efficiency of multifunctional nanocarriers is reported in the literature, they presently suffer challenges related to reproducibility, scalability, and long-term stability. The complexity of such nanocarriers introduces variability in synthesis and performance, which can hinder large-scale production and clinical translation.

### 7.3. Regulatory Framework and Clinical Adoption

For any drug development process, regulatory and translational challenges pose significant barriers to clinical adoption. These parameters are viewed as more critical than technical and biological considerations. Typically, development of nanomedicine-based therapies involves rigorous evaluation of pharmacokinetics, biodistribution, long-term toxicity, and immunogenicity. Although regulatory bodies such as the U.S. Food and Drug Administration and the European Medicines Agency have established general guidelines for nanotherapeutics, standardized frameworks tailored specifically to multifunctional nanoparticle systems are still evolving [86,119]. Variations in nanoparticle composition, surface modification, and manufacturing processes further complicate quality control, reproducibility and regulatory approval.

For successful applications and large-scale production of nanoplatforms, it is imperative to design a standard protocol that is reproducible, scalable and in compliance with the GMP guidelines. The important variables of the design include size, distribution and morphology. In addition, the composition of calcium bases and its crystalline structure is important in the drug design process [134]. In addition, a dataset of their characteristics should be prepared including information on surface chemistry, drug-loading efficiency, encapsulation stability, Ca^2+^ release kinetics, sterility, endotoxin content, residual solvent levels, and physicochemical stability during storage [135,136]. Characterization of nanoplatforms should be done preferably using validated and advanced analytical techniques. Once the nanoplatforms are successfully designed, they should be manufactured in batches along with in process quality controls. Throughout the production system, a standard purification method should be used [135,136]. Following these systematic steps, production of high quality and therapeutically efficient nanoplatforms can be ensured [137].

### 7.4. Biodistribution, Clearance, and Long-Term Biosafety of Calcium-Based Nanoplatforms

The calcium-based nanoplatforms show high efficiency due to their ability to selectively accumulate inside tumor cells [39]. Additionally, their efficiency is influenced by factors such as biodistribution, biodegradability, interaction with non-target sites and ease of clearance from the circulatory system [39]. Regarding the introduction of nanoparticles in the human body through systemic administration, its stability and efficacy depend mainly on the physicochemical properties (particle size, morphology, surface charge, composition, and surface functionalization) [138]. Altogether, these attributes influence circulation time of particles, protein corona formation, interactions with macromolecules and susceptibility to phagocytic attack by the mononuclear phagocyte system (MPS) [138]. The resulting accumulation of nanoparticles in the liver and spleen may lead to off-target effects [138]. Although the enhanced permeability and retention (EPR) effect generally favors selective targeting of tumor cells, the reported extent of accumulation is varied in tumor types and patients [139]. This limits the efficiency of nanoparticle delivery at selective sites and increases the chance for off-target accumulation [139].

The mechanism of action of calcium-based nanoplatforms such as calcium phosphate (CaP), calcium carbonate (CaCO_3_), and calcium peroxide (CaO_2_) generally involves undergoing gradual degradation in the acidic tumor microenvironment to release calcium, phosphate and carbonate ions [37,39,47]. These ions interfere with the cells physiological metabolic processes and restrict their growth [37,47]. Subsequently, the soluble particles are eliminated through renal excretion, while incompletely degraded larger particles are either sequestered by liver and spleen, internalized by macrophages or undergo hepatobiliary clearance [140,141]. These processes along with repeated administration can lead to persistence of nanoparticles in the system, prolong tissue exposure and alteration of biodistribution patterns. Hence, there is a need for in depth studies on pharmacokinetic and biodistribution of nanoplatforms.

Although the calcium-based nanoplatforms are highly efficient, there are several major safety concerns associated with them. For instance, premature degradation of these platforms can lead to uncontrolled calcium release outside the cellular matrix [142,143]. It may also lead to ectopic calcification due to mineral deposition in non-target healthy tissues [142,143]. This condition may further progress to nephrocalcinosis, vascular calcification, impaired organ function, or thrombotic complications following heavy depositions in the kidneys, heart, lungs and other soft tissues [143,144,145,146,147]. Hence, design of calcium-based therapeutics is critical to ensure calcium overload following degradation at target site that can induce endothelial dysfunction, coagulation, and tumor vascular collapse [148]. At the same time, efficient therapeutic design is essential for preventing systemic toxicity [148].

The current preclinical studies generally report efficient biocompatibility of biodegradable calcium-based nanomaterials. Precisely, the calcium-based inorganic biodegradable nanomaterials (CIBNs) such as calcium phosphate, hydroxyapatite, calcium carbonate, and calcium sulfate are gaining importance as promising biomedical materials due to their physiological relevance [149,150]. Since these materials are naturally present in the human body at nanoscale dimensions, they are very likely to show biocompatibility [149,150]. Additionally, their stability favors controlled release designs of pH-dependent tumor targeting, and feasibility favors large-scale applications [151]. Furthermore, their biodegradability ensures safe elimination from the body [39]. Consequently, CIBNs show versatile applications in processes such as tissue engineering, bone/teeth regeneration, systemic drug delivery and cancer therapy [39,151]. Despite the suitability of calcium-based nanotherapeutics, several challenges persist in their clinical translation. Few examples include instances of colloidal instability and nanoparticle aggregation [152]. Also, scaling a reproducible manufacturing process is challenging and standard protocols remain undefined [152]. In addition, there are several gaps in our current understanding of long-term pharmacokinetic and toxicological profiles of calcium-based nanoplatforms. Precisely, most of the preclinical models described so far describe the effect of nanoparticles following acute or sub-acute exposure periods [153]. Although these models successfully demonstrate short-term biocompatibility, prolonged circulation, and no toxicity on oral administration, conclusive facts and opinions regarding factors such as chronic biodistribution, repeated-dose administration, long-term clearance and cumulative toxicity remain unstated [153]. In summary, the mechanisms of nanoparticle biodistribution and their pharmacokinetic characteristics that altogether govern the in vivo fate of several nanomaterials are not well understood [154]. Since these are important determinants of efficacy and toxicity, there is a need for systematic and long-term investigations of these factors in in vivo models before nanoparticle-based processes can be effectively translated into clinical processes [154,155,156].

To address the major gaps in our current understanding, future investigations should focus on developing biodegradable precision formulations with properties such as selective tumor delivery and stimuli-responsive release systems to prevent or minimize off-target accumulation. Additionally, developing standardized datasets with information regarding pharmacokinetics, biodistribution, metabolism, excretion, and long-term toxicology should be prioritized [39,88]. In addition, the development of standard protocols for quantitative assessment of ectopic calcification in organs may be extremely helpful in evaluating nanotherapeutic dynamics in in vivo systems and determining their safety profile.

## 8. Conclusions

Nanoparticle-mediated calcium therapy is a promising approach to cancer treatment. It offers drug-free mechanisms for rapid and irreversible shutdown of tumor vasculature, and hence prevents development of drug resistance in tumor cells. The advances in nanotechnology further allow precision and enhanced synergy with combination therapies. Moreover, the localized delivery and tumor cell destruction occurring through calcium-dependent coagulation, endothelial damage, and ischemia improves selectivity. Considering the immense potential of nanoparticle-mediated calcium therapy and the advances in nanotechnology, the present challenges related to controlled calcium release and delivery efficiency can be expected to be overcome in near future. For ensuring systemic safety and regulatory concerns, however, large-scale clinical trials are essential. With continued interdisciplinary research and clinical validation, calcium-based vascular disruption holds strong potential as a cost-effective and impactful component of the next-generation cancer therapies.

## Figures and Tables

**Table 2:** Advantages and challenges of calcium-mediated vascular disruption.

Aspect	Advantages	Challenges	References
**Vascular Targeting**	Rapid and potentially irreversible vessel occlusion	Risk of premature or off-target Ca^2+^ release leading to unintended thrombosis or vascular injury	[81,82,98,115]
**Tumor Selectivity**	Exploits the EPR effect, acidic tumor microenvironment, and abnormal vasculature	Tumor heterogeneity, variable extracellular pH, and limited EPR-mediated accumulation in human tumors may reduce nanoparticle delivery and Ca^2+^ release	[39,86,88,130]
**Physiological Calcium Regulation**	Localized Ca^2+^ release induces intracellular calcium overload in tumor cells	Interactions with physiological calcium regulation systems may influence Ca^2+^ bioavailability and therapeutic efficacy	[39,102]
**Resistance Profile**	Reduced susceptibility to conventional drug resistance through physicochemical vascular occlusion	Hypoxia-driven adaptive mechanisms, including vessel co-option and vascular mimicry, may reduce long-term efficacy	[75,76,77]
**Therapeutic Synergy**	Compatible with chemotherapy, phototherapy, sonodynamic therapy, immunotherapy, and thermal ablation	Multifunctional nanoplatforms require optimization of release kinetics, formulation stability, and manufacturing reproducibility	[102,131,132]
**Safety Profile**	Localized delivery may reduce systemic toxicity	Off-target Ca^2+^ release may induce ectopic calcification, vascular injury, and long-term biosafety concerns	[81,82]
**Pharmacokinetics and Biodistribution**	Stimuli-responsive nanoplatforms improve targeted delivery	Long-term biodistribution, clearance, organ accumulation, and interactions with systemic calcium homeostasis remain insufficiently characterized	[39,86,102]
**Clinical Translation**	Cost-effective and biocompatible calcium-based materials	Lack of standardized manufacturing, limited human data, and absence of Phase I clinical trials for calcium-overload-mediated vascular disruption	[39,86]
**Imaging Integration**	Enables real-time monitoring of nanoparticle distribution and therapeutic response	Increased regulatory complexity for multifunctional theranostic platforms	[103]
**Nanotechnology Benefits**	Controlled release and improved targeting	Manufacturing reproducibility, large-scale production, and long-term stability remain challenging	[86,119]

## Data Availability

No new data were created or analyzed in this study. Data sharing is not applicable to this article.
